# Factors associated with the risk perception of COVID-19 infection and severe illness: A cross-sectional study in Japan

**DOI:** 10.1016/j.ssmph.2022.101105

**Published:** 2022-04-26

**Authors:** Megumi Adachi, Michio Murakami, Daisuke Yoneoka, Takayuki Kawashima, Masahiro Hashizume, Haruka Sakamoto, Akifumi Eguchi, Cyrus Ghaznavi, Stuart Gilmour, Satoshi Kaneko, Hiroyuki Kunishima, Keiko Maruyama-Sakurai, Yuta Tanoue, Yoshiko Yamamoto, Hiroaki Miyata, Shuhei Nomura

**Affiliations:** a Department of Health Policy and Management, School of Medicine, Keio University, Tokyo, Japan; b Department of Global Health Policy, Graduate School of Medicine, The University of Tokyo, Tokyo, Japan; c Division of Scientific Information and Public Policy, Center for Infectious Disease Education and Research, Osaka University, Osaka, Japan; d Tokyo Foundation for Policy Research, Tokyo, Japan; e Infectious Disease Surveillance Center, National Institute of Infectious Diseases, Tokyo, Japan; f Department of Mathematical and Computing Science, Tokyo Institute of Technology, Tokyo, Japan; g Department of Hygiene and Public Health, Tokyo Women's Medical University, Tokyo, Japan; h Center for Preventive Medical Sciences, Chiba University, Chiba, Japan; i Medical Education Program, Washington University School of Medicine in St Louis, Saint Louis, USA; j Graduate School of Public Health, St. Luke's International University, Tokyo, Japan; k Department of Ecoepidemiology, Institute of Tropical Medicine, Nagasaki University, Nagasaki, Japan; l Department of Infectious Diseases, St. Marianna University School of Medicine, Kanagawa, Japan; m Institute for Business and Finance, Waseda University, Tokyo, Japan; n National Center for Child Health and Development, Tokyo, Japan

**Keywords:** COVID-19, Risk perception, Japan, Infection, Severe illness

## Abstract

Understanding COVID-19 risk perception may help inform public health messaging aimed at encouraging preventive measures and improving countermeasures against the pandemic. We conducted an online survey of 29,708 Japanese adults in February 2021 and estimated the associations between COVID-19 risk perception and a broad array of individual factors. Two logistic regressions were constructed to estimate factors associated with the risk perception of COVID-19 (defined as responding that one might become infected within the next 6 months), and of severe illness among those who responded that they might become infected (defined as responding that one would become severely ill). After adjusting for covariates, those with a higher perceived risk of the COVID-19 vaccine had higher odds of risk perception for both infection and severe illness. Interestingly, those with higher odds of risk perception of being infected were more likely to report obtaining their information from healthcare workers whereas those with lower odds were more likely to report obtaining their information from the Internet or the government; those with lower odds of risk perception of being severely ill were more likely to report obtaining their information from the Internet. The higher the trust level in the government as a COVID-19 information source, the lower the odds of both risk perception of being infected and becoming severely ill. The higher the trust levels in social networking services as a COVID-19 information source, the higher the odds of risk perception of becoming severely ill. Public health messaging should address the factors identified in our study.

## Highlights

• Perceived risks of a COVID-19 vaccine was related to risk perception of COVID-19 infection and severe illness.
• Getting information from healthcare workers was related to higher risk perception of infection.
• Higher trust in SNS was related to higher risk perception of COVID-19 of severe illness.

## 1 Introduction

The coronavirus disease 2019 (COVID-19) pandemic has spread across the world. The outbreak has since caused more than 255 million cases and over 5.1 million deaths globally as of November 19, 2021 (World Health Organization, 2020a, World Health Organization, 2020b, World Health Organization, 2020c). Having safe and effective vaccines against COVID-19 is one of the most important means of ending the pandemic (World Health Organization, 2020a, World Health Organization, 2020b, World Health Organization, 2020c), and COVID-19 vaccination campaigns are underway in many countries (World Health Organization, 2020a, World Health Organization, 2020b, World Health Organization, 2020c). As of November 22, 2021, 41.62% of the world population and 76.21% of the Japanese population have received two vaccination doses (Japan is ranked eighth in the world) (Our World in Data); however, vaccination coverage remains very low in many low- and middle-income countries, and in some high-income countries, vaccine hesitancy has resulted in continued high infection rates, even as vaccination rates have increased (Omer et al., 2021). It is expected to take some time for the entire world to achieve high levels of vaccine coverage.

In addition, the effectiveness of the COVID-19 vaccine against COVID-19 infection and onset has been shown to decline over several months (Tartof et al., 2021), and a third dose of vaccination is being offered in some countries (Centers for Disease Control and Prevention (CDC), 2021, Ministry of Health, National Health Service (NHS)). As of April 2022, some countries also have begun offering fourth doses of vaccination (Bar-On et al., 2022). Several studies have warned that if various activity restrictions are lifted before vaccination rates are high enough, the number of cases will rise again (Centers for Disease Control and Prevention (CDC), Public Health England, World Health Organization, 2020a, World Health Organization, 2020b, World Health Organization, 2020c). Therefore, vaccination will continue to be an important tool to contain the pandemic, but it is necessary to continue preventive measures while promoting vaccination.

Among the many challenges associated with the COVID-19 pandemic, maintaining and improving the practice of preventive measures and adherence to behavior restrictions remains one of the most important tasks. Adherence to these policies largely depend on any given individual's risk perception of COVID-19 (Brewer et al., 2007; Myhre et al., 2020; Setbon & Raude, 2010; Weinstein et al., 2007). Risk perception is a central component of health-specific behavioral theories, such as the health belief model and protection motivation theory (Brewer et al., 2007; Tagini, Brugnera, Ferrucci, Mazzocco, Compare, et al., 2021). People feel risk when they perceive an event as uncontrolled, catastrophic, new, and unknown (Slovic, 1987). It is known that there are two basic ways that people perceive risk: “risk as feelings” and “risk as analysis.” The risk as feeling model is a fast, instinctive, and intuitive reaction to danger, while the analytical model is logical, reasoned, relatively slow, and effortful (Slovic et al., 2004). Previous studies have shown that risk perception varies cognitively, emotionally, socially, culturally, and between individuals (Af WÅhlberg, 2001, Douglas & Wildavsky, 1983, Joffe, 2003, Kasperson et al., 1988, Leiserowitz, 2006, Loewenstein et al., 2001, Sjöberg, 2002, Slovic, 2010, Slovic et al., 1982, Van der Linden, 2015, 2017, Wildavsky and Dake).

Risk perception is associated with various factors, including sociodemographic characteristics. Many previous studies investigated sociodemographic determinants of COVID-19 risk perception. Women, the elderly, wealthy people, and those with underlying diseases were more likely to have a higher risk perception of COVID-19 (Eyeberu et al., 2021; He et al., 2021, Monge-Rodríguez et al., 2021; Zeballos Rivas, Lopez Jaldin, Nina Canaviri, Portugal Escalante, Alanes Fernandez, et al., 2021). Second, risk perception is associated with psychological factors. Few studies have attempted to identify the psychological factors that characterize people with high or low risk perception of COVID-19 (Dryhurst et al., 2020; Kalam et al., 2021; Lim et al., 2021, Lin et al., 2020, Lin et al., 2020; Monge-Rodríguez et al., 2021; Tagini, Brugnera, Ferrucci, Mazzocco, Pievani, et al., 2021). Third, vaccine-related factors also drive risk perception. However, the association between perceptions of risk and the benefits of a COVID-19 vaccine is not yet known. This study assessed variables such as perceived norms about a COVID-19 vaccine and perceived benefits of the COVID-19 vaccine. Finally, factors related to information sources are also linked to risk perception. Several studies reported that use of or trust in information sources were associated with risk perception of COVID-19 (Dryhurst et al., 2020; Lim et al., 2021, Lin, Tu, & Beitsch, 2020; Monge-Rodríguez et al., 2021; Tagini, Brugnera, Ferrucci, Mazzocco, Compare, et al., 2021). The relationship between COVID-19 risk perception, information sources, and trust in them varies from country to country and region to region (Lim et al., 2021; Monge-Rodríguez et al., 2021). Knowledge about these factors can provide an opportunity to tailor public health messages aimed at improving preventive measures and increasing adherence by explicitly targeting specific populations and addressing population-specific factors relating to COVID-19 risk perception (Pan American Health Organization, 2020; Paulik et al., 2020; Sauer et al., 2021; World Health Organization, 2018).

Though most of the recent evidence on COVID-19 risk perception has been generated in Europe and the United States, there is little evidence for other regions, such as Asia, including Japan (Dryhurst et al., 2020, Monge-Rodríguez et al., 2021; Tagini et al., 2021a, 2021b, Zeballos Rivas, Lopez Jaldin, Nina Canaviri, Portugal Escalante, Alanes Fernandez, et al., 2021). In addition, previous studies were conducted during the early stages of the pandemic with relatively small sample sizes and limited representativeness (Dryhurst et al., 2020; Shiina et al., 2020).

We conducted an online survey of lifestyles during the pandemic in a large sample that is fairly representative of Japan's population during February to March 2021, just after the third wave of infections and before COVID-19 vaccination of the general public had begun. We measured an individual's risk perception of COVID-19 by asking about self-reported predictions of COVID-19 infection and severe illness within the next six months. The objective of this study was to estimate the association between the perceived risk of COVID-19 infection and severe illness and sociodemographic factors, psychological and vaccine-related characteristics, and the level of trust in various sources of information.

## 2 Methods

### 2.1 Study population

Details of the survey used in this study have been described elsewhere (Nomura et al., 2021). Briefly, the survey participants were those who were registered with a web research company (Cross Marketing Inc.). The panel members were aged 20 years or older and able to answer online questionnaires in Japanese. The survey incentive was “points” given in return for answering questionnaires, with the number of points based on the number of responses. Panel members could use these points to purchase products and services from partner companies. In this survey, we recruited approximately 30,000 participants through a quota sampling method: subjects were recruited such that the demographics of the survey population based on age (at the time of the survey), gender, and prefecture matched data obtained from the 2015 National Census. Recruitment was on a first-come, first-served basis, and the recruitment period closed when the preset number of respondents was reached. The survey opened on February 26, 2021 and reached its target on March 4, 2021. Respondents were required to answer all questions in order to receive their points, and thus there was no missing data.

### 2.2 Surveys

In developing the questionnaire used in the online survey, we thoroughly reviewed previous literature on similar topics (Lin, Tu, & Beitsch, 2020, Robinson et al., 2021). The details of the survey questionnaire can be found elsewhere (Nomura et al., 2021), but in brief, the questionnaire consisted of three parts: part I asked about the sociodemographic characteristics of participants as well as health-related topics, including health literacy; part II asked about psychological and vaccine-related characteristics (e.g., perceived risks of the COVID-19 vaccine, perceived norms about the COVID-19 vaccine); part III was concerned with information sources that the participants usually use to obtain COVID-19 information and their level of trust in those sources. Health literacy here refers to the ability to obtain, read, understand, and use information about health care in order to make good health decisions and follow treatment instructions (Institute of Medicine, 2012). All questions were closed-ended questions and took the form of single or multiple responses, such as binary (yes or no), nominal and ordinal scales, and Likert scale questions. Unless otherwise noted, respondent information is current at the time of survey response.

### 2.3 Outcome measures

Perceived risk of COVID-19 was measured with the question: ‘what is your best guess as to whether you will get COVID-19 within the next 6 months?” Response options were “I don't think I will get COVID-19,” “I think I will get a mild case of COVID-19,” “I think I will get severely ill from COVID-19,” and “I have already had COVID-19.” The last option does not assess risk perception, so those who chose it (i.e., those who have already experienced COVID-19 infection by the time of the survey) were excluded from the present study. Based on the responses to the remaining three options, then, individuals were classified into three risk perception groups. Hereafter, these are referred to as groups 1, 2, and 3.

### 2.4 Other variables

All questions are outlined in the resulting tables. The variables considered in our survey were derived directly from the existing evidence base on behavior and risk perception regarding COVID-19, including vaccination acceptance, and are summarized below (Lin, Tu, & Beitsch, 2020, Robinson et al., 2021).

Regarding sociodemographic variables, we asked respondents about their gender, prefecture of residence, highest level of education, occupation, annual household income in 2020, household size, marital status, and the impact of the COVID-19 pandemic on their lives. Health-related variables included self-reported health status, experience with COVID-19 testing, presence of underlying diseases, whether they lived with elderly family members or those with underlying diseases, experience of COVID-19 infection among those close to them, and health literacy. Finally, we asked respondents about their history of influenza and routine immunizations to assess their general attitude toward vaccination. All variables were treated as categorical with the exception of highest level of education, annual household income in 2020, and household size.

Regarding psychological and vaccine-related variables, we investigated the perceived risks and benefits of vaccines against COVID-19 as well as the beliefs and perceived norms of respondents. Perceived norms here refer to whether they believe they should be vaccinated if others are vaccinated. We also examined the level of trust in the scientists involved in the development of the COVID-19 vaccine, the public authorities that approved the vaccine, and the healthcare providers who administer the vaccines. These psychological and vaccine-related characteristics were measured with single items rather than validated scales in order to reduce respondent burden and ease of interpretation (Bowling, 2005). These variables were treated as categorical variables.

Regarding sources of COVID-19 information and the level of trust in these sources, we considered 30 options, including several types of healthcare professionals, news media by medium, social networking services (SNS), and government bodies. The levels of trust in information sources were evaluated on a Likert scale but were treated as continuous variables, ranging from zero (no trust at all) to three (extremely trustworthy), which allowed for the calculation of the mean and standard deviation (SD) of the scores for each source.

### 2.5 Data analysis

Descriptive information was compared between the three risk perception groups. The number of people (%) or the mean (SD) was calculated to show the characteristics of the study population. Chi-squared tests and one-way ANOVA were used to calculate differences among the risk perceptions groups. The purpose of these descriptive analyses was to show the characteristics of the participants as a basic tabulation, and F-statistic or post-hoc tests were not computed.

Odds ratios (OR) with 95% confidence intervals (CI) of COVID-19 risk perceptions were estimated using multivariable logistic regression with a backward-forward stepwise variable selection method, removing those with p ≥ 0.1 and adding terms with p < 0.05. We built two logistic regression models: Model 1 estimated the odds of answering ‘I think I will get a mild case of COVID-19' or ‘I think I will get severely ill from COVID-19’ among all participants, where group 1 was coded as 0 and group 2 and group 3 were coded as 1; and Model 2 estimated the odds of answering ‘I think I will get severely ill from COVID-19’ among group 2 and group 3 (those who believed they would become infected), where group 2 was coded as 0 and group 3 was coded as 1. In other words, Model 1 can estimate the odds of perceived high risk of infection within the next 6 months, and Model 2 can estimate the odds of perceived risk of severe illness within the next 6 months among those who believe they are at risk of infection. Unless otherwise noted, these odds are hereafter referred to simply as the odds of risk perception of infection and risk perception of severe illness.

Our survey investigated a very large number of variables and response options; if all of them were taken into account in the regression, the model would suffer from overfitting and multicollinearity problems. Hence, we tried to reduce the number of variables by integrating the response options of several variables. For example, place of residence was reclassified from 47 prefectures to 6 regions: Hokkaido and Tohoku, Kanto, Chubu, Kansai, Chugoku and Shikoku, and Kyushu and Okinawa. The sources of information and their level of trust were integrated as follows: doctors, nurses, pharmacists, veterinarians, and dentists were reclassified as “healthcare workers; ” magazines and books as “books and magazines; ” newspapers, television, and radio as “television, radio, and newspapers; ” information from Internet news sites and search engines (Google, Yahoo, etc.) as “the Internet; ” LINE, Facebook, Twitter, YouTube, and Tik Tok as “SNS; ” local governments and governments as “governments; ” friends and family as “friends and family; ” scientists, researchers, and pharmaceutical companies as “researchers.” When reclassifying for binary questions (i.e., information sources), 1 was coded if any of the options were chosen, and for Likert scale questions (i.e., trust level), the average value among the options was treated as the result for individual responses. Cronbach's alpha coefficients were used as internal consistency estimates for the reclassified trust level variables.

Two-sided p-values less than 0.05 were considered statistically significant. All analyses were performed using STATA/BE version 17. After excluding those who have experienced COVID-19 infection by the time of the survey (n = 298) and those who answered “other” for gender (as it is difficult to make stable estimations due to the small number of respondents) (n = 53), a total of 29,708 participants were included in this study.

## 3 Result

### 3.1 Sociodemographic and health-related characteristics

The participants’ sociodemographic and health-related characteristics are presented in Table 1. The mean age was 52.43 years (SD 16.35) and ranged from 20 to 99. Approximately half of the participants (52.03%) were women. More than 40% of the participants had graduated from university. The most common types of occupation were homemaker (22.46%), manufacturing (10.18%), and services (not elsewhere classified) (8.51%). More than half of the participants (55.48%) were in group 1, around one-third of the participants (32.32%) were in group 2, and 12.20% of the participants were in group 3. There were statistically significant differences between the three risk perception groups in the distribution of all sociodemographic and health-related variables.

**Table 1:** Sociodemographic and health-related characteristics of the participants.

	Group 1	Group 2	Group 3	Total
	N (% in column) = 16,481 (55.48%)	N (% in column) = 9603 (32.32%)	N (% in column) = 3624 (12.20%)	N = 29,708
**Sociodemographic characteristics**				
Age (years)				
Mean (SD)	55.22 (16.10)	46.86 (15.47)	54.49 (16.02)	52.43 (16.35)
Gender (SA)				
Women	8740 (53.03)	4849 (50.49)	1867 (51.52)	15,456 (52.03)
Men	7741 (46.97)	4754 (49.51)	1757 (48.48)	14,252 (47.97)
Region (SA)				
Hokkaido and Tohoku	1948 (11.82)	1058 (11.02)	429 (11.84)	3435 (11.56)
Hokkaido	913 (5.54)	487 (5.07)	204 (5.63)	1604 (5.40)
Aomori	151 (0.92)	86 (0.90)	22 (0.61)	259 (0.87)
Iwate	129 (0.78)	91 (0.95)	34 (0.94)	254 (0.85)
Miyagi	325 (1.97)	175 (1.82)	87 (2.40)	587 (1.98)
Akita	124 (0.75)	60 (0.62)	15 (0.41)	199 (0.67)
Yamagata	119 (0.72)	63 (0.66)	25 (0.69)	207 (0.70)
Fukushima	187 (1.13)	96 (1.00)	42 (1.16)	325 (1.09)
Kanto	5364 (32.55)	3345 (34.83)	1308 (36.09)	10,017 (33.72)
Ibaraki	277 (1.68)	161 (1.68)	49 (1.35)	487 (1.64)
Tochigi	162 (0.98)	101 (1.05)	40 (1.10)	303 (1.02)
Gunma	165 (1.00)	79 (0.82)	34 (0.94)	278 (0.94)
Saitama	868 (5.27)	507 (5.28)	189 (5.22)	1564 (5.26)
Chiba	752 (4.56)	436 (4.54)	185 (5.10)	1373 (4.62)
Tokyo	1829 (11.10)	1270 (13.23)	524 (14.46)	3623 (12.20)
Kanagawa	1311 (7.95)	791 (8.24)	287 (7.92)	2389 (8.04)
Chubu	3109 (18.86)	1710 (17.81)	619 (17.08)	5438 (18.30)
Niigata	349 (2.12)	173 (1.80)	67 (1.85)	589 (1.98)
Toyama	146 (0.89)	84 (0.87)	30 (0.83)	260 (0.88)
Ishikawa	158 (0.96)	91 (0.95)	22 (0.61)	271 (0.91)
Fukui	96 (0.58)	40 (0.42)	19 (0.52)	155 (0.52)
Yamanashi	103 (0.62)	50 (0.52)	23 (0.63)	176 (0.59)
Nagano	291 (1.77)	149 (1.55)	66 (1.82)	506 (1.70)
Gifu	228 (1.38)	125 (1.30)	44 (1.21)	397 (1.34)
Shizuoka	404 (2.45)	224 (2.33)	74 (2.04)	702 (2.36)
Aichi	1092 (6.63)	653 (6.80)	236 (6.51)	1981 (6.67)
Mie	242 (1.47)	121 (1.26)	38 (1.05)	401 (1.35)
Kansai	2601 (15.78)	1625 (16.92)	567 (15.65)	4793 (16.13)
Shiga	126 (0.76)	89 (0.93)	34 (0.94)	249 (0.84)
Kyoto	311 (1.89)	205 (2.13)	78 (2.15)	594 (2.00)
Osaka	1172 (7.11)	743 (7.74)	235 (6.48)	2150 (7.24)
Hyogo	741 (4.50)	442 (4.60)	168 (4.64)	1351 (4.55)
Nara	176 (1.07)	105 (1.09)	37 (1.02)	318 (1.07)
Wakayama	75 (0.46)	41 (0.43)	15 (0.41)	131 (0.44)
Chugoku and Shikoku	1556 (9.44)	821 (8.55)	288 (7.95)	2665 (8.97)
Tottori	65 (0.39)	43 (0.45)	12 (0.33)	120 (0.40)
Shimane	65 (0.39)	49 (0.51)	21 (0.58)	135 (0.45)
Okayama	296 (1.80)	165 (1.72)	55 (1.52)	516 (1.74)
Hiroshima	410 (2.49)	226 (2.35)	79 (2.18)	715 (2.41)
Yamaguchi	162 (0.98)	75 (0.78)	25 (0.69)	262 (0.88)
Tokushima	96 (0.58)	53 (0.55)	21 (0.58)	170 (0.57)
Kagawa	166 (1.01)	79 (0.82)	22 (0.61)	267 (0.90)
Ehime	221 (1.34)	96 (1.00)	36 (0.99)	353 (1.19)
Kochi	75 (0.46)	35 (0.36)	17 (0.47)	127 (0.43)
Kyusyu and Okinawa	1903 (11.55)	1044 (10.87)	413 (11.40)	3360 (11.31)
Fukuoka	867 (5.26)	517 (5.38)	207 (5.71)	1591 (5.36)
Saga	94 (0.57)	53 (0.55)	23 (0.63)	170 (0.57)
Nagasaki	188 (1.14)	71 (0.74)	30 (0.83)	289 (0.97)
Kumamoto	221 (1.34)	105 (1.09)	46 (1.27)	372 (1.25)
Oita	117 (0.71)	79 (0.82)	26 (0.72)	222 (0.75)
Miyazaki	128 (0.78)	45 (0.47)	25 (0.69)	198 (0.67)
Kagoshima	162 (0.98)	93 (0.97)	28 (0.77)	283 (0.95)
Okinawa	126 (0.76)	81 (0.84)	28 (0.77)	235 (0.79)
Highest educational level (SA)				
Middle school	475 (2.88)	216 (2.25)	130 (3.59)	821 (2.76)
High school	5828 (35.36)	2908 (30.28)	1327 (36.62)	10,063 (33.87)
Junior college	3193 (19.37)	1906 (19.85)	685 (18.90)	5784 (19.47)
University	6327 (38.39)	4102 (42.72)	1326 (36.59)	11,755 (39.57)
Graduate school (master's course)	532 (3.23)	381 (3.97)	107 (2.95)	1020 (3.43)
Graduate school (doctoral course)	126 (0.76)	90 (0.94)	49 (1.35)	265 (0.89)
Occupation type (SA)				
Agriculture, forestry and fisheries	117 (0.71)	51 (0.53)	31 (0.86)	199 (0.67)
Construction	414 (2.51)	339 (3.53)	112 (3.09)	865 (2.91)
Manufacturing	1436 (8.71)	1224 (12.75)	365 (10.07)	3025 (10.18)
Information and communications	472 (2.86)	332 (3.46)	92 (2.54)	896 (3.02)
Transportation and postal services	388 (2.35)	329 (3.43)	92 (2.54)	809 (2.72)
Wholesale and retail trade	1026 (6.23)	723 (7.53)	218 (6.02)	1967 (6.62)
Finance and insurance	328 (1.99)	272 (2.83)	79 (2.18)	679 (2.29)
Real estate and goods rental and leasing	203 (1.23)	152 (1.58)	78 (2.15)	433 (1.46)
Scientific research, professional and technical services	212 (1.29)	147 (1.53)	46 (1.27)	405 (1.36)
Accommodations, food and beverage services	341 (2.07)	244 (2.54)	67 (1.85)	652 (2.19)
Living-related and personal services and amusement services	237 (1.44)	183 (1.91)	57 (1.57)	477 (1.61)
Education and learning support	527 (3.20)	409 (4.26)	95 (2.62)	1031 (3.47)
Medical healthcare and welfare	889 (5.39)	761 (7.92)	216 (5.96)	1866 (6.28)
Combined services	105 (0.64)	95 (0.99)	34 (0.94)	234 (0.79)
Services (not elsewhere classified)	1306 (7.92)	909 (9.47)	313 (8.64)	2528 (8.51)
Public service (not elsewhere classified)	474 (2.88)	349 (3.63)	99 (2.73)	922 (3.10)
Students	278 (1.69)	298 (3.10)	43 (1.19)	619 (2.08)
Homemaker	4204 (25.51)	1592 (16.58)	877 (24.20)	6673 (22.46)
Others	3524 (21.38)	1194 (12.43)	710 (19.59)	5428 (18.27)
Annual household income in 2020 (million JPY; thousand USD) (SA)				
–1); −9.2)	1117 (6.78)	590 (6.14)	332 (9.16)	2039 (6.86)
[1–2); [9.2–18.4)	1566 (9.50)	721 (7.51)	402 (11.09)	2689 (9.05)
[2–3); [18.4–27.6)	2395 (14.53)	1186 (12.35)	541 (14.93)	4122 (13.88)
[3–4); [27.6–36.8)	2687 (16.30)	1346 (14.02)	570 (15.73)	4603 (15.49)
[4–5); [36.8–46.0)	2024 (12.28)	1320 (13.75)	480 (13.25)	3824 (12.87)
[5–6); [46–55.2)	1638 (9.94)	1039 (10.82)	321 (8.86)	2998 (10.09)
[6–7); [55.2–64.4)	1189 (7.21)	808 (8.41)	245 (6.76)	2242 (7.55)
[7–8); [64.4–73.6)	1056 (6.41)	714 (7.44)	190 (5.24)	1960 (6.60)
[8–9); [73.6–82.8)	701 (4.25)	476 (4.96)	128 (3.53)	1305 (4.39)
[9–10); [82.8–92.0)	2108 (12.79)	1403 (14.61)	415 (11.45)	3926 (13.22)
Household size including respondent (SA)				
1	2997 (18.18)	1835 (19.11)	691 (19.07)	5523 (18.59)
2	6731 (40.84)	2922 (30.43)	1414 (39.02)	11,067 (37.25)
3	3735 (22.66)	2396 (24.95)	815 (22.49)	6946 (23.38)
4	2109 (12.80)	1733 (18.05)	462 (12.75)	4304 (14.49)
5	619 (3.76)	503 (5.24)	164 (4.53)	1286 (4.33)
More than 6	290 (1.76)	214 (2.23)	78 (2.15)	582 (1.96)
Marital size (SA)				
Married (including de facto marriage)	10,237 (62.11)	5484 (57.11)	2212 (61.04)	17,933 (60.36)
Not married (without partner)	3546 (21.52)	2669 (27.79)	788 (21.74)	7003 (23.57)
Not married (with a partner)	753 (4.57)	738 (7.69)	196 (5.41)	1687 (5.68)
Widowed	784 (4.76)	214 (2.23)	166 (4.58)	1164 (3.92)
Divorced	1161 (7.04)	498 (5.19)	262 (7.23)	1921 (6.47)
To what extent did the COVID-19 pandemic affect your life, within the past year? (SA)				
Not at all	1281 (7.77)	439 (4.57)	132 (3.64)	1852 (6.23)
Not much	4710 (28.58)	2481 (25.84)	661 (18.24)	7852 (26.43)
Somewhat	8246 (50.03)	5082 (52.92)	1900 (52.43)	15,228 (51.26)
Quite a lot	2244 (13.62)	1601 (16.67)	931 (25.69)	4776 (16.08)
**Health-related characteristics, including health literacy**				
Self-reported health status (SA)				
Very good	5101 (30.95)	2880 (29.99)	600 (16.56)	8581 (28.88)
Good	4986 (30.25)	3143 (32.73)	913 (25.19)	9042 (30.44)
Fair	4637 (28.14)	2633 (27.42)	1170 (32.28)	8440 (28.41)
Poor	1449 (8.79)	824 (8.58)	723 (19.95)	2996 (10.08)
Very poor	308 (1.87)	123 (1.28)	218 (6.02)	649 (2.18)
Have you ever received a COVID-19 test? (MA)				
No	15,322 (92.97)	8693 (90.52)	3223 (88.93)	27,238 (91.69)
Yes – PCR test	789 (4.79)	613 (6.38)	233 (6.43)	1635 (5.50)
Yes – antigen test	119 (0.72)	139 (1.45)	79 (2.18)	337 (1.13)
Yes – antibody testing	140 (0.85)	107 (1.11)	52 (1.43)	299 (1.01)
Yes – unsure about either of the three	183 (1.11)	126 (1.31)	76 (2.10)	385 (1.30)
Presence of underlying diseases (e.g. diabetes, heart failure, respiratory disease, COPD, etc., or on dialysis, or using immunosuppressive or anticancer drugs) (SA)	2238 (13.58)	708 (7.37)	992 (27.37)	3938 (13.26)
Living with family members who are elderly or have underlying diseases (SA)	2778 (16.86)	1277 (13.30)	787 (21.72)	4842 (16.30)
Do you receive an influenza vaccine? (SA)				
Every year	5873 (35.63)	3575 (37.23)	1559 (43.02)	11,007 (37.05)
Every few years	2807 (17.03)	1952 (20.33)	684 (18.87)	5443 (18.32)
Rarely or never	7801 (47.33)	4076 (42.45)	1381 (38.11)	13,258 (44.63)
Did you receive on routine immunization? (SA)				
All	3313 (20.10)	2126 (22.14)	818 (22.57)	6257 (21.06)
Partially	2857 (17.34)	1748 (18.20)	754 (20.81)	5359 (18.04)
None	7051 (42.78)	3800 (39.57)	1379 (38.05)	12,230 (41.17)
Not sure	3260 (19.78)	1929 (20.09)	673 (18.57)	5862 (19.73)
Has anyone close to you ever been infected with COVID-19? (MA)				
Family or friends	431 (2.62)	360 (3.75)	139 (3.84)	930 (3.13)
Colleagues at work	537 (3.26)	720 (7.50)	197 (5.44)	1454 (4.89)
No/I don't know.	15,539 (94.28)	8567 (89.21)	3300 (91.06)	27,406 (92.25)
How confident are you filling out medical forms by yourself?” (health literacy) (SA)				
Not at all	187 (1.13)	140 (1.46)	94 (2.59)	421 (1.42)
A little bit	554 (3.36)	508 (5.29)	307 (8.47)	1369 (4.61)
Somewhat	3404 (20.65)	2325 (24.21)	1051 (29.00)	6780 (22.82)
Extremely	7385 (44.81)	4202 (43.76)	1401 (38.66)	12,988 (43.72)
Quite a bit	4951 (30.04)	2428 (25.28)	771 (21.27)	8150 (27.43)

SD: Standard deviation, SA: Single answer, MA: Multiple answer.

### 3.2 Psychological and vaccine-related characteristics

Psychological and vaccine-related characteristics of the participants are shown in Table 2. 61.25% of the participants felt vaguely anxious about COVID-19. 13.82% of the participants felt that the disadvantages of a COVID-19 vaccine were large or very large. 58.60% of the participants felt that the benefits of a COVID-19 vaccine were large or very large. The differences between the three risk perception groups were statistically significant for all psychological and vaccine-related characteristics.

**Table 2:** Psychological and vaccine-related characteristics of the participants.

	Group 1	Group 2	Group 3	Total
	N (% in column) = 16,481 (55.48%)	N (% in column) = 9603 (32.32%)	N (% in column) = 3624 (12.20%)	N = 29,708
How anxious are you about COVID-19? (SA)				
Not at all anxious	1841 (11.17)	609 (6.34)	63 (1.74)	2513 (8.46)
Vaguely anxious	10,687 (64.84)	6122 (63.75)	1388 (38.30)	18,197 (61.25)
Have a clear sense of anxiety	3221 (19.54)	2351 (24.48)	1497 (41.31)	7069 (23.79)
Feel fear and have anxiety	732 (4.44)	521 (5.43)	676 (18.65)	1929 (6.49)
How do you think the disadvantages of the COVID-19 vaccine are? (SA) – perceived risks of a COVID-19 vaccine				
Very small	1614 (9.79)	639 (6.65)	250 (6.90)	2503 (8.43)
Small	5889 (35.73)	2959 (30.81)	1010 (27.87)	9858 (33.18)
Medium	6912 (41.94)	4673 (48.66)	1657 (45.72)	13,242 (44.57)
Large	1621 (9.84)	1144 (11.91)	571 (15.76)	3336 (11.23)
Very large	445 (2.70)	188 (1.96)	136 (3.75)	769 (2.59)
How do you feel are the benefits of the COVID-19 vaccine? (SA) – perceived benefits of a COVID-19 vaccine				
Very small	508 (3.08)	214 (2.23)	93 (2.57)	815 (2.74)
Small	1011 (6.13)	742 (7.73)	232 (6.40)	1985 (6.68)
Medium	4993 (30.30)	3271 (34.06)	1234 (34.05)	9498 (31.97)
Large	8179 (49.63)	4556 (47.44)	1627 (44.90)	14,362 (48.34)
Very large	1790 (10.86)	820 (8.54)	438 (12.09)	3048 (10.26)
Do you think getting a COVID-19 vaccine will ease your anxiety? (SA) – perceived benefits of a COVID-19 vaccine				
Strongly disagree	780 (4.73)	375 (3.91)	185 (5.10)	1340 (4.51)
Disagree	2503 (15.19)	1491 (15.53)	563 (15.54)	4557 (15.34)
Neither agree nor disagree	5317 (32.26)	3433 (35.75)	1256 (34.66)	10,006 (33.68)
Agree	7126 (43.24)	3932 (40.95)	1413 (38.99)	12,471 (41.98)
Strongly agree	755 (4.58)	372 (3.87)	207 (5.71)	1334 (4.49)
If others have been vaccinated against COVID-19, do you think you should be vaccinated as well? (SA) – perceived norms about a COVID-19 vaccine				
Strongly disagree	763 (4.63)	288 (3.00)	99 (2.73)	1150 (3.87)
Disagree	1815 (11.01)	923 (9.61)	294 (8.11)	3032 (10.21)
Neither agree nor disagree	4530 (27.49)	3078 (32.05)	1108 (30.57)	8716 (29.34)
Agree	7399 (44.89)	4256 (44.32)	1508 (41.61)	13,163 (44.31)
Strongly agree	1974 (11.98)	1058 (11.02)	615 (16.97)	3647 (12.28)
Do you trust scientists in the field of vaccine development for COVID-19? (SA) – trusts about COVID-19 vaccine				
Strongly distrust	365 (2.21)	165 (1.72)	81 (2.24)	611 (2.06)
Distrust	901 (5.47)	607 (6.32)	236 (6.51)	1744 (5.87)
Neutral	6009 (36.46)	3960 (41.24)	1464 (40.40)	11,433 (38.48)
Trust	8502 (51.59)	4497 (46.83)	1665 (45.94)	14,664 (49.36)
Strongly trust	704 (4.27)	374 (3.89)	178 (4.91)	1256 (4.23)
Do you trust the public authorities to approve vaccines for COVID-19? (SA) – trusts about COVID-19 vaccine				
Strongly distrust	578 (3.51)	298 (3.10)	153 (4.22)	1029 (3.46)
Distrust	1478 (8.97)	1078 (11.23)	436 (12.03)	2992 (10.07)
Neutral	6368 (38.64)	4063 (42.31)	1507 (41.58)	11,938 (40.18)
Trust	7548 (45.80)	3891 (40.52)	1404 (38.74)	12,843 (43.23)
Strongly trust	509 (3.09)	273 (2.84)	124 (3.42)	906 (3.05)
Do you trust your healthcare provider about vaccination against COVID-19? (SA) – trusts about COVID-19 vaccine				
Strongly distrust	316 (1.92)	142 (1.48)	74 (2.04)	532 (1.79)
Distrust	633 (3.84)	471 (4.90)	180 (4.97)	1284 (4.32)
Neutral	4461 (27.07)	2978 (31.01)	1151 (31.76)	8590 (28.91)
Trust	10,011 (60.74)	5332 (55.52)	1954 (53.92)	17,297 (58.22)
Strongly trust	1060 (6.43)	680 (7.08)	265 (7.31)	2005 (6.75)
Do you think that healthcare workers and employees of elderly care facilities should be vaccinated? (SA) – belief about COVID-19 vaccine				
Yes	12,839 (77.90)	7225 (75.24)	2744 (75.72)	22,808 (76.77)
No	550 (3.34)	372 (3.87)	148 (4.08)	1070 (3.60)
Can't say either	3092 (18.76)	2006 (20.89)	732 (20.20)	5830 (19.62)

SA: Single answer, MA: Multiple answer.

### 3.3 Information sources about COVID-19 and trust levels

The most common information sources were television (82.18%), followed by Internet news sites (53.71%) and newspapers (35.67%) (Table 3). 14.06% of the participants obtained information from healthcare workers. 17.00% of the participants obtained information from social networking sites (SNS), with the most common SNS being Twitter (7.94%), LINE (7.09%), and You Tube (5.38%). There were statistically significant differences in the distribution of information sources among the three risk perception groups.

**Table 3:** Participants’ use of information sources of COVID-19.

	Group 1	Group 2	Group 3	Total
	N (% in column) = 16,481 (55.48%)	N (% in column) = 9603 (32.32%)	N (% in column) = 3624 (12.20%)	N = 29,708
From what sources do you get information about COVID-19? (MA)				
Healthcare workers	2040 (12.38)	1429 (14.88)	708 (19.54)	4177 (14.06)
Physicians	1717 (10.42)	1114 (11.60)	598 (16.50)	3429 (11.54)
Nurses	592 (3.59)	469 (4.88)	226 (6.24)	1287 (4.33)
Pharmacists	272 (1.65)	204 (2.12)	109 (3.01)	585 (1.97)
Veterinarians	20 (0.12)	27 (0.28)	17 (0.47)	64 (0.22)
Dentists	155 (0.94)	93 (0.97)	53 (1.46)	301 (1.01)
Health fairs and events	107 (0.65)	99 (1.03)	51 (1.41)	257 (0.87)
Books and magazines	928 (5.63)	622 (6.48)	253 (6.98)	1803 (6.07)
Books	371 (2.25)	307 (3.20)	124 (3.42)	802 (2.70)
Magazines	752 (4.56)	474 (4.94)	197 (5.44)	1423 (4.79)
Scientific literature	182 (1.10)	151 (1.57)	64 (1.77)	397 (1.34)
Television, radio and newspaper	14,390 (87.31)	7984 (83.14)	3044 (84.00)	25,418 (85.56)
Television	13,852 (84.05)	7639 (79.55)	2922 (80.63)	24,413 (82.18)
Radio	2018 (12.24)	1153 (12.01)	511 (14.10)	3682 (12.39)
Newspapers	6417 (38.94)	2931 (30.52)	1249 (34.46)	10,597 (35.67)
The Internet	9785 (59.37)	5615 (58.47)	1969 (54.33)	17,369 (58.47)
Internet news sites	9109 (55.27)	5054 (52.63)	1793 (49.48)	15,956 (53.71)
Search engines (Google, Yahoo, etc.)	3366 (20.42)	2230 (23.22)	772 (21.30)	6368 (21.44)
SNS	2611 (15.84)	1845 (19.21)	595 (16.42)	5051 (17.00)
LINE	1093 (6.63)	752 (7.83)	261 (7.20)	2106 (7.09)
Facebook	304 (1.84)	201 (2.09)	95 (2.62)	600 (2.02)
Twitter	1107 (6.72)	970 (10.10)	282 (7.78)	2359 (7.94)
Instagram	154 (0.93)	202 (2.10)	66 (1.82)	422 (1.42)
YouTube	857 (5.20)	550 (5.73)	192 (5.30)	1599 (5.38)
Tik Tok	45 (0.27)	37 (0.39)	24 (0.66)	106 (0.36)
Medical information sites	334 (2.03)	296 (3.08)	118 (3.26)	748 (2.52)
Blogs or web pages of celebrities and famous people	295 (1.79)	201 (2.09)	68 (1.88)	564 (1.90)
Governments	5894 (35.76)	2921 (30.42)	1198 (33.06)	10,013 (33.70)
Local authorities such as prefectures and municipalities	5151 (31.25)	2459 (25.61)	1034 (28.53)	8644 (29.10)
Government	3136 (19.03)	1673 (17.42)	652 (17.99)	5461 (18.38)
Medical task-force advising the government, known as the Novel Coronavirus Expert Meeting (re-established as Novel Coronavirus Infectious Disease Control Subcommittee in July 2020)	1428 (8.66)	724 (7.54)	300 (8.28)	2452 (8.25)
Family and friends	3824 (23.20)	1824 (18.99)	759 (20.94)	6407 (21.57)
Friends	2186 (13.26)	1154 (12.02)	450 (12.42)	3790 (12.76)
Family members	3159 (19.17)	1516 (15.79)	629 (17.36)	5304 (17.85)
Researchers	525 (3.19)	313 (3.26)	136 (3.75)	974 (3.28)
Scientists and researchers	450 (2.73)	250 (2.60)	111 (3.06)	811 (2.73)
Pharmaceutical companies	140 (0.85)	107 (1.11)	51 (1.41)	298 (1.00)
Other companies	406 (2.46)	211 (2.20)	118 (3.26)	735 (2.47)

MA: Multiple answer, SNS: Social networking services.

The trust levels in information sources are presented in Table 4. All Cronbach's alpha coefficients for the reclassified variables were above 0.80. Physicians were the most trusted information source for all participants (mean 2.73, SD 0.75), followed by nurses (mean 2.60, SD 0.73) and pharmacists (mean 2.48, SD 0.73). The mean trust levels in SNS was 1.57 (SD 0.62) (LINE: 1.71, SD 0.70; Facebook: 1.56 SD 0.68; Twitter: 1.57 SD 0.69; Instagram: 1.53 SD 0.67; You Tube: 1.59 SD 0.69; Tik Tok: 1.43, SD 0.65), which was the lowest mean trust level of all information sources. There were statistically significant differences in the distribution of most of the trust levels in information sources among the three risk perception groups.

**Table 4:** Trust levels of information sources.

	Group 1	Group 2	Group 3	Total	Cronbach's alpha
	N = 16,481 (55.48%)	N = 9603 (32.32%)	N = 3624 (12.20%)	N = 29,708	
	Mean (SD)	Mean (SD)	Mean (SD)	Mean (SD)	
Healthcare workers	2.42 (0.62)	2.42 (0.61)	2.44 (0.66)	2.42 (0.62)	0.86
Physicians	2.74 (0.74)	2.72 (0.75)	2.74 (0.78)	2.73 (0.75)	
Nurses	2.60 (0.72)	2.60 (0.73)	2.61 (0.76)	2.60 (0.73)	
Pharmacists	2.47 (0.72)	2.48 (0.72)	2.49 (0.76)	2.48 (0.73)	
Veterinarians	2.06 (0.75)	2.09 (0.76)	2.10 (0.79)	2.08 (0.76)	
Dentists	2.20 (0.75)	2.21 (0.75)	2.25 (0.79)	2.21 (0.75)	
Health fairs and events	1.99 (0.69)	1.99 (0.71)	2.00 (0.76)	1.99 (0.71)	
Books and magazines	1.98 (0.65)	2.00 (0.66)	1.99 (0.69)	1.99 (0.66)	0.89
Magazines	1.94 (0.69)	1.95 (0.70)	1.94 (0.74)	1.94 (0.70)	
Books	2.03 (0.67)	2.05 (0.69)	2.03 (0.72)	2.04 (0.68)	
Scientific literature	2.38 (0.73)	2.36 (0.72)	2.35 (0.75)	2.37 (0.73)	
Television, radio, and newspapers	2.24 (0.65)	2.17 (0.64)	2.22 (0.66)	2.22 (0.65)	0.88
Newspapers	2.31 (0.74)	2.23 (0.73)	2.26 (0.75)	2.28 (0.74)	
Television	2.26 (0.73)	2.17 (0.72)	2.24 (0.76)	2.23 (0.73)	
Radio	2.16 (0.69)	2.11 (0.69)	2.15 (0.72)	2.14 (0.69)	
The Internet	2.06 (0.62)	2.04 (0.62)	2.05 (0.67)	2.05 (0.63)	0.87
Internet news sites	2.09 (0.66)	2.06 (0.67)	2.07 (0.71)	2.08 (0.67)	
Search engines (Google, Yahoo, etc.)	2.03 (0.66)	2.01 (0.66)	2.04 (0.71)	2.02 (0.67)	
SNS	1.55 (0.60)	1.59 (0.62)	1.59 (0.67)	1.57 (0.62)	0.96
LINE	1.70 (0.69)	1.73 (0.70)	1.73 (0.76)	1.71 (0.70)	
Facebook	1.54 (0.66)	1.59 (0.68)	1.59 (0.73)	1.56 (0.68)	
Twitter	1.54 (0.67)	1.60 (0.69)	1.59 (0.74)	1.57 (0.69)	
Instagram	1.51 (0.66)	1.56 (0.68)	1.56 (0.73)	1.53 (0.67)	
YouTube	1.57 (0.67)	1.61 (0.69)	1.62 (0.74)	1.59 (0.69)	
Tik Tok	1.41 (0.63)	1.45 (0.66)	1.47 (0.71)	1.43 (0.65)	
Medical information sites	2.19 (0.72)	2.17 (0.73)	2.15 (0.77)	2.18 (0.73)	
Blogs or web pages of celebrities and famous people	1.62 (0.68)	1.66 (0.70)	1.65 (0.73)	1.64 (0.69)	
Governments	2.39 (0.71)	2.32 (0.69)	2.30 (0.73)	2.36 (0.70)	0.89
Local authorities such as prefectures and municipalities	2.43 (0.74)	2.36 (0.74)	2.36 (0.78)	2.40 (0.75)	
Government	2.26 (0.78)	2.21 (0.77)	2.15 (0.81)	2.23 (0.78)	
Medical task-force advising the government, known as the Novel Coronavirus Expert Meeting (re-established as Novel Coronavirus Infectious Disease Control Subcommittee in July 2020)	2.48 (0.81)	2.40 (0.78)	2.39 (0.83)	2.45 (0.80)	
Family and friends	2.08 (0.65)	2.06 (0.65)	2.09 (0.70)	2.08 (0.66)	0.84
Friends	1.97 (0.67)	1.98 (0.68)	1.97 (0.73)	1.98 (0.68)	
Family members	2.19 (0.73)	2.14 (0.72)	2.21 (0.78)	2.18 (0.73)	
Researchers	2.28 (0.68)	2.22 (0.67)	2.24 (0.72)	2.25 (0.69)	0.86
Scientists and researchers	2.35 (0.75)	2.28 (0.73)	2.31 (0.78)	2.32 (0.75)	
Pharmaceutical companies	2.20 (0.71)	2.17 (0.71)	2.17 (0.75)	2.19 (0.72)	
Other companies	1.89 (0.67)	1.91 (0.67)	1.91 (0.72)	1.90 (0.68)	

SD: Standard deviation, SNS: Social networking services.

### 3.4 Factors associated with risk perception of infection within the next 6 months (Model 1)

The results of Model 1 are presented in Table 5. After adjusting for covariates, among sociodemographic and health-related variables, age, region, occupation, annual household income, household size, marital status, the impact of the COVID-19 pandemic on their lives, self-rated health status, experience with COVID-19 testing, experience of COVID-19 infection among those close to respondents, health literacy, history of influenza vaccinations, and history of routine immunizations were found to be associated with risk perception of infection. Those with higher age (OR 0.98, 95% CI 0.98–0.98) and higher annual household income (OR 0.98, 95% CI 0.97–0.99) had statistically significantly lower odds. Compared to the Kanto region, most regions had lower odds (Hokkaido and Tohoku: OR 0.90; 95% CI 0.83–0.98, Chubu: OR 0.87; 95% CI 0.81–0.94, Chugoku and Shikoku: OR 0.88; 95% CI 0.81–0.97, Kyushu and Okinawa: OR 0.91; 95% CI 0.84–0.99). Participants who reported that the COVID-19 pandemic had a greater impact on their lives had higher odds than those who did not. Participants who had someone close to them infected with COVID-19 had higher odds. The odds were also lower for those who do not or rarely receive an influenza vaccine than those who do every year (OR 0.81, 95% CI 0.76–0.86). Furthermore, the odds were lower for those who had not received routine immunizations compared to those who had (OR 0.91, 95% CI 0.84–0.98). However, the odds were not statistically significant for those who had underlying diseases.

**Table 5:** Factors associated with the risk perception of COVID-19 infection.

	Odds ratio	95% Confidence Interval	p-value
**Sociodemographic characteristics**			
Age	0.98	0.98–0.98	<0.001
Gender (SA)			
Women (reference)	1.00	NA	NA
Men	1.03	0.97–1.09	0.30
Region (SA)			
Hokkaido and Tohoku	0.90	0.83–0.98	<0.05
Kanto (reference)	1.00	NA	NA
Chubu	0.87	0.81–0.94	<0.001
Kansai	1.01	0.94–1.09	0.78
Chugoku and Shikoku	0.88	0.81–0.97	<0.01
Kyusyu and Okinawa	0.91	0.84–0.99	<0.05
Highest educational level (SA)	1.00	0.98–1.03	0.84
Occupation type (SA)			
Agriculture, forestry and fisheries	0.84	0.61–1.15	0.27
Construction	1.17	0.98–1.39	0.08
Manufacturing	1.11	0.98–1.26	0.09
Information and communications	0.86	0.73–1.02	0.09
Transportation and postal services	1.11	0.93–1.33	0.23
Wholesale and retail trade	0.99	0.87–1.14	0.91
Finance and insurance	1.05	0.87–1.26	0.62
Real estate and goods rental and leasing	1.38	1.11–1.73	<0.01
Scientific research, professional and technical services	1.07	0.85–1.35	0.54
Accommodations, food and beverage services	0.88	0.73–1.06	0.18
Living-related and personal services and amusement services	1.02	0.83–1.26	0.84
Education and learning support	1.10	0.94–1.29	0.24
Medical healthcare and welfare (reference)	1.00	NA	NA
Combined services	1.20	0.90–1.60	0.22
Services (not elsewhere classified)	1.04	0.91–1.18	0.56
Public service (not elsewhere classified)	1.05	0.89–1.25	0.56
Students	0.75	0.61–0.91	<0.01
Homemaker	0.80	0.71–0.90	<0.001
Others	0.76	0.67–0.86	<0.001
Annual household income in 2020 (million JPY; thousand USD) (SA)	0.98	0.97–0.99	<0.001
Household size including respondent (SA)	1.02	1.00–1.05	<0.05
Marital size (SA)			
Married (including de facto marriage) (reference)	1.00	NA	NA
Not married (without partner)	0.88	0.82–0.95	<0.01
Not married (with a partner)	0.95	0.84–1.06	0.36
Widowed	0.92	0.81–1.06	0.24
Divorced	0.86	0.78–0.96	<0.01
To what extent did the COVID-19 pandemic affect your life, within the past year? (SA)			
Not at all (reference)	1.00	NA	NA
Not much	1.63	1.45–1.83	<0.001
Somewhat	2.04	1.82–2.28	<0.001
Quite a lot	2.41	2.13–2.72	<0.001
**Health-related characteristics, including health literacy**			
Self-reported health status (SA)			
Very good	0.77	0.72–0.83	<0.001
Good	0.98	0.92–1.04	0.51
Fair (reference)	1.00	NA	NA
Poor	1.25	1.15–1.37	<0.001
Very poor	1.25	1.05–1.48	<0.05
Have you ever received a COVID-19 test? (MA)			
Yes – PCR test	0.98	0.88–1.10	0.75
Yes – antigen test	1.43	1.12–1.82	<0.01
Yes – antibody testing	0.95	0.74–1.22	0.70
Yes – unsure about either of the three	1.15	0.93–1.42	0.20
Presence of underlying diseases (e.g. diabetes, heart failure, respiratory disease, COPD, etc., or on dialysis, or using immunosuppressive or anticancer drugs) (SA)	0.99	0.92–1.07	0.89
Do you receive an influenza vaccine? (SA)			
Every year (reference)	1.00	NA	NA
Every few years	1.01	0.94–1.08	0.78
Rarely or never	0.81	0.76–0.86	<0.001
Did you receive on routine immunization? (SA)			
All (reference)	1.00	NA	NA
Partially	1.02	0.94–1.11	0.61
None	0.91	0.84–0.98	<0.05
Not sure	0.83	0.76–0.89	<0.001
Has anyone close to you ever been infected with COVID-19? (MA)			
Family or friends	1.22	1.06–1.41	<0.01
Colleagues at work	1.49	1.32–1.68	<0.001
How confident are you filling out medical forms by yourself?” (health literacy) (SA)			
Not at all	1.17	0.94–1.45	0.15
A little bit	1.31	1.15–1.48	<0.001
Somewhat (reference)	1.00	NA	NA
Extremely	0.87	0.82–0.93	<0.001
Quite a bit	0.83	0.77–0.90	<0.001
**Vaccine-related characteristics**			
How do you think the disadvantages of the COVID-19 vaccine are? (SA) – perceived risks of a COVID-19 vaccine			
Very small	0.65	0.58–0.72	<0.001
Small	0.82	0.77–0.88	<0.001
Medium (reference)	1.00	NA	NA
Large	1.13	1.04–1.23	<0.01
Very large	0.89	0.74–1.07	0.20
How do you feel are the benefits of the COVID-19 vaccine? (SA) – perceived benefits of a COVID-19 vaccine			
Very small	0.87	0.72–1.05	0.14
Small	1.13	1.01–1.26	<0.05
Medium (reference)	1.00	NA	NA
Large	1.02	0.95–1.10	0.54
Very large	0.87	0.78–0.98	<0.05
If others have been vaccinated against COVID-19, do you think you should be vaccinated as well? (SA) – perceived norms about a COVID-19 vaccine			
Strongly disagree	0.54	0.46–0.64	<0.001
Disagree	0.71	0.65–0.78	<0.001
Neither agree nor disagree (reference)	1.00	NA	NA
Agree	1.09	1.02–1.17	<0.01
Strongly agree	1.38	1.24–1.53	<0.001
Do you trust the public authorities to approve vaccines for COVID-19? (SA) – trusts about COVID-19 vaccine			
Strongly distrust	1.15	0.95–1.39	0.16
Distrust	1.19	1.08–1.31	<0.001
Neither agree nor disagree (reference)	1.00	NA	NA
Trust	0.95	0.89–1.01	0.12
Strongly trust	0.86	0.71–1.03	0.10
Do you trust your healthcare provider about vaccination against COVID-19? (SA) – trusts about COVID-19 vaccine			
Strongly distrust	1.02	0.79–1.33	0.85
Distrust	1.10	0.96–1.26	0.17
Neither agree nor disagree (reference)	1.00	NA	NA
Trust	0.94	0.87–1.01	0.08
Strongly trust	1.12	0.97–1.29	0.12
**Information sources (MA)**			
Healthcare workers	1.26	1.17–1.35	<0.001
Books and Magazines	1.14	1.03–1.26	<0.05
The Internet	0.91	0.86–0.95	<0.001
Medical information sites	1.38	1.18–1.62	<0.001
Governments	0.94	0.88–0.99	<0.05
Family and friends	0.85	0.80–0.90	<0.001
Other companies	0.75	0.64–0.88	<0.01
**Trust levels in information sources (MA)**			
Healthcare workers	1.16	1.10–1.22	<0.001
Books and Magazines	1.07	1.01–1.13	<0.05
Television, radio and newspaper	0.85	0.80–0.90	<0.001
Governments	0.90	0.85–0.95	<0.001
Other companies	1.06	1.01–1.11	<0.05

SA: Single answer, MA: Multiple answer, Healthcare workers: Physicians, nurses, pharmacists, veterinarians, and dentists, the Internet: Internet news sites and search engines (Google, Yahoo, etc.), Governments: Local authorities such as prefectures and municipalities, government, Medical task-force advising the government, known as the Novel Coronavirus Expert Meeting (re-established as Novel Coronavirus Infectious Disease Control Subcommittee in July 2020). Age, highest educational level, annual household income in 2020, household size including respondent were treated as continuous variables.

In terms of vaccine-related characteristics, COVID-19 vaccine-related perceived risk, benefit, norms, and trust were identified as factors associated with perceived risk of infection. The odds were lower for those who had lower perceived risk of the COVID-19 vaccine or higher perceived benefits of the COVID-19 vaccine. For the perceived norms about the COVID-19 vaccine, assessed by the question ‘if others have been vaccinated against COVID-19, do you think you should be vaccinated as well?’, the odds were higher for those who agree than those who disagree. Those who did not trust the public authorities who approved the COVID-19 vaccine were more likely to have higher odds of risk perception of infection.

For information sources, participants who obtain COVID-19 information from healthcare workers (OR 1.26, 95% CI 1.17–1.35), books and magazines (OR 1.14, 95% CI 1.03–1.26), and medical information sites (OR 1.38, 95% CI 1.18–1.62) had higher odds than those who do not. On the other hand, those who use the Internet (OR 0.91, 95% CI 0.86–0.95) and those who obtain information from governments (OR 0.94, 95% CI 0.88–0.99) or friends or family members (OR 0.85, 95% CI 0.80–0.90) had lower odds than those who do not. Participants with higher trust levels in healthcare workers (OR 1.16, 95% CI 1.10–1.22) and books and magazines (OR 1.07, 95% CI 1.01–1.13) had higher odds. Also, participants who had higher trust in television, radio, and newspapers (OR 0.85, 95% CI 0.80–0.90) and governments (OR 0.90, 95% CI 0.85–0.95) had lower odds.

### 3.5 Factors associated with risk perception of severe illness within the next 6 months (Model 2)

The results of Model 2 are presented in Table 6. After adjusting for covariates, among sociodemographic and health-related variables, age, region, occupation type, annual household income, the impact of the COVID-19 pandemic on their lives, self-reported health status, experience with COVID-19 testing, presence of underlying diseases, experience of COVID-19 infection among those close to them, health literary, and history of influenza vaccinations were statistically significantly related to risk perception of severe illness. Those with higher age (OR 1.03, 95% CI 1.02–1.03) had higher odds. Compared to the Kanto region, most regions had lower odds (Hokkaido and Tohoku: OR 0.81; 95% CI 0.70–0.93, Chubu: OR 0.82; 95% CI 0.73–0.93, Kansai: OR 0.83; 95% CI 0.73–0.94, Chugoku and Shikoku: OR 0.79; 95% CI 0.67–0.93). Participants who reported that the COVID-19 pandemic had a large impact on their lives had higher odds than those who reported that it had none (OR 1.81, 95% CI 1.43–2.29). Odds were also higher for those who have underlying diseases (OR 3.17, 95% CI 2.80–3.58) and those who received the influenza vaccine annually than for those who did not.

**Table 6:** Factors associated with risk perception of severe illness.

	Odds ratio	95% Confidence Intervals	p-value
**Sociodemographic characteristics**			
Age	1.03	1.02–1.03	<0.001
Gender (SA)			
Women (reference)	1.00	NA	NA
Men	1.01	0.92–1.12	0.81
Region (SA)			
Hokkaido and Tohoku	0.81	0.70–0.93	<0.01
Kanto	1.00	NA	NA
Chubu	0.82	0.73–0.93	<0.01
Kansai	0.83	0.73–0.94	<0.01
Chugoku and Shikoku	0.79	0.67–0.93	<0.01
Kyusyu and Okinawa	0.88	0.76–1.02	0.09
Highest educational level (SA)	0.99	0.94–1.03	0.56
Occupation type (SA)			
Agriculture, forestry and fisheries	1.85	1.10–3.11	<0.05
Construction	1.25	0.94–1.67	0.12
Manufacturing	1.19	0.97–1.48	0.10
Information and communications	1.13	0.84–1.53	0.42
Transportation and postal services	0.96	0.71–1.30	0.80
Wholesale and retail trade	1.10	0.87–1.40	0.41
Finance and insurance	1.12	0.81–1.54	0.49
Real estate and goods rental and leasing	1.54	1.09–2.17	<0.05
Scientific research, professional and technical services	1.21	0.81–1.79	0.35
Accommodations, food and beverage services	1.00	0.71–1.40	0.99
Living-related and personal services and amusement services	1.10	0.77–1.59	0.60
Education and learning support	0.86	0.64–1.15	0.31
Medical healthcare and welfare (reference)	1.00	NA	NA
Combined services	1.12	0.71–1.78	0.62
Services (not elsewhere classified)	1.05	0.84–1.31	0.65
Public service (not elsewhere classified)	1.14	0.85–1.53	0.39
Students	0.99	0.67–1.44	0.94
Homemaker	1.41	1.16–1.73	<0.01
Others	1.13	0.92–1.39	0.25
Annual household income in 2020 (million JPY; thousand USD) (SA)	0.96	0.94–0.97	<0.001
To what extent did the COVID-19 pandemic affect your life, within the past year? (SA)			
Not at all (reference)	1.00	NA	NA
Not much	0.86	0.68–1.09	0.21
Somewhat	1.23	0.99–1.55	0.07
Quite a lot	1.81	1.43–2.29	<0.001
**Health-related characteristics, including health literacy**			
Self-reported health status (SA)			
Very good	0.65	0.57–0.73	<0.001
Good	0.75	0.67–0.84	<0.001
Fair (reference)	1.00	NA	NA
Poor	1.52	1.33–1.74	<0.001
Very poor	2.77	2.13–3.59	<0.001
Have you ever received a COVID-19 test? (MA)			
Yes – PCR test	0.97	0.81–1.17	0.76
Yes – antigen test	1.47	1.07–2.03	<0.05
Yes – antibody testing	1.23	0.84–1.81	0.29
Yes – unsure about either of the three	1.40	1.02–1.93	<0.05
Presence of underlying diseases (e.g. diabetes, heart failure, respiratory disease, COPD, etc., or on dialysis, or using immunosuppressive or anticancer drugs) (SA)	3.17	2.80–3.58	<0.001
Living with family members who are elderly or have underlying diseases (SA)	1.11	0.99–1.24	0.09
Do you receive an influenza vaccine? (SA)			
Every year (reference)	1.00	NA	NA
Every few years	0.87	0.77–0.98	<0.05
Rarely or never	0.82	0.74–0.91	<0.001
Has anyone close to you ever been infected with COVID-19? (MA)			
Family or friends	0.93	0.75–1.16	0.54
Colleagues at work	0.81	0.67–0.97	<0.05
How confident are you filling out medical forms by yourself?” (health literacy) (SA)			
Not at all	1.33	0.97–1.82	0.08
A little bit	1.20	1.00–1.44	<0.05
Somewhat (reference)	1.00	NA	NA
Extremely	0.81	0.72–0.90	<0.001
Quite a bit	0.79	0.70–0.91	<0.01
**Vaccine-related characteristics**			
How do you think the disadvantages of the COVID-19 vaccine are? (SA) – perceived risks of a COVID-19 vaccine			
Very small	0.79	0.64–0.97	<0.05
Small	0.86	0.76–0.96	<0.01
Medium (reference)	1.00	NA	NA
Large	1.38	1.20–1.57	<0.001
Very large	1.52	1.14–2.02	<0.01
How do you feel are the benefits of the COVID-19 vaccine? (SA) – perceived benefits of a COVID-19 vaccine			
Very small	0.97	0.70–1.34	0.83
Small	0.84	0.69–1.01	0.07
Medium (reference)	1.00	NA	NA
Large	0.92	0.82–1.04	0.17
Very large	1.15	0.94–1.41	0.16
Do you think getting a COVID-19 vaccine will ease your anxiety? (SA) – perceived benefits of a COVID-19 vaccine			
Strongly disagree	1.44	1.11–1.86	<0.01
Disagree	1.04	0.91–1.19	0.59
Neither agree nor disagree (reference)	1.00	NA	NA
Agree	0.95	0.85–1.07	0.40
Strongly agree	1.03	0.80–1.32	0.84
If others have been vaccinated against COVID-19, do you think you should be vaccinated as well? (SA) – perceived norms about a COVID-19 vaccine			
Strongly disagree	0.63	0.45–0.87	<0.01
Disagree	0.88	0.74–1.04	0.13
Neither agree nor disagree (reference)	1.00	NA	NA
Agree	0.99	0.88–1.11	0.86
Strongly agree	1.48	1.24–1.76	<0.001
**Information sources (MA)**			
The Internet	0.87	0.80–0.95	<0.01
Other companies	1.72	1.34–2.22	<0.001
**Trust levels in information sources (MA)**			
Healthcare workers	1.07	0.99–1.17	0.10
Television, radio and newspaper	1.14	1.04–1.25	<0.01
SNS	1.15	1.06–1.25	<0.01
Medical information sites	0.90	0.84–0.98	<0.01
Governments	0.85	0.78–0.93	<0.001

SA: Single answer, MA: Multiple answer, The Internet: Internet news sites and search engines (Google, Yahoo, etc.), SNS: Social networking services, Governments: Local authorities such as prefectures and municipalities, government, Medical task-force advising the government, known as the Novel Coronavirus Expert Meeting (re-established as Novel Coronavirus Infectious Disease Control Subcommittee in July 2020). Age, highest educational level, annual household income in 2020 were treated as continuous variables.

For vaccine-related characteristics, the perceived risks, benefits and norms of the COVID-19 vaccine were identified to be factors associated with risk perception of severe illness. The odds were lower for those who had lower perceived risks of the COVID-19 vaccine, and higher for those who had lower perceived benefits of the COVID-19 vaccine. Regarding perceived norms about the COVID-19 vaccine, those who agreed had higher odds than those who did not agree.

Regarding COVID-19 information sources, those who use internet news sites and search engines had lower odds (OR 0.87, 95% CI 0.80–0.95) than those who do not. Higher trust levels in television, radio, and newspapers and SNS were associated with higher odds, while higher trust levels in medical information sites and governments were associated with lower odds.

## 4 Discussion

Little is known about the determinants of perceived risk of COVID-19 infection in Japan (Shiina et al., 2020). Based on an online survey with 30,000 respondents aged 20 years and older conducted just before vaccination began in the country, we found that 55.48% of respondents did not believe they would be infected with COVID-19 in the next 6 months, 32.32% believed they would get a mild case of COVID-19, and 12.20% believed they would get seriously ill from COVID-19. The risk perception of infection and severe illness were statistically significantly more likely to be associated with sociodemographic, health-related, and vaccine-related characteristics of participants, as well as information sources about COVID-19 and levels of trust in them.

### 4.1 Factors associated with perceived risk of infection within the next 6 months

This study showed that higher age was statistically significantly associated with lower risk perception of infection. This finding supports those of a previous study conducted in March 2020 during the early stages of the pandemic in the United States, which found that while older people perceived a greater infection-fatality risk than younger people, they also believed that they were at lower risk of becoming infected (Bruine de Bruin, 2021). This may be consistent with the fact that while being older is indeed a risk factor for COVID-19 severe illness, older people tend to be less responsive to everyday stressors (Carstensen et al., 2000; Neubauer et al., 2019). Another explanation may be that older people are less socially active than their younger counterparts and therefore were less likely to have a perceived risk of infection. As for gender, we found that there was no statistically significant association between gender and risk perception of infection. The statistical significance of the perceived risk of COVID-19 infection varied among previous studies; some reported higher levels for women (Chisty et al., 2021; de Zwart et al., 2007; Ding et al., 2020, Mansilla Domínguez et al., 2020, Zeballos Rivas, Lopez Jaldin, Nina Canaviri, Portugal Escalante, Alanes Fernández, et al., 2021), while others reported no significant difference between genders (Barr et al., 2008; Chan et al., 2020). In addition, we found that the experience of COVID-19 infection among those close to respondents was statistically significantly associated with higher risk perception. Domínguez et al. (2020) found a similar relationship between the risk perception of COVID−19 infection in Spain with contact and direct experience with the infection in family, friends, or coworkers (Mansilla Domínguez et al., 2020).

Our study also found that those who perceived less risk from the COVID-19 vaccine had were less likely to perceive a risk of infection; those who perceived more benefit from the vaccine had a lower perceived risk of infection. These findings may be a consequence of people's awareness of the fact that vaccination lowers the risk of infection. In March 2021, when our survey was conducted, the effectiveness of the COVID-19 vaccine in reducing the risk of infection and serious illness due to COVID-19, such as hospitalization and mortality, had been reported by the US, the UK, Israel, and other countries (Britton et al., 2021, Public Health England, 2021; Rinott et al., 2021). The awareness created from those reports may have lowered risk perception of infection among participants. According to a study conducted in Bangladesh in early 2021, people who received the COVID-19 vaccine were more likely than those who did not to cite the benefits of the vaccine with respect to reducing the risk of COVID-19 infection and benefits related to their livelihood, resuming economic activities, and returning to normalcy (Kalam et al., 2021).

The risk perception of COVID-19 infection was more likely to be higher among those who relied on healthcare workers, books and magazines, and medical information sites as sources of information about COVID-19. It was also more likely to be higher among those who trusted medical professionals, books, and magazines as sources of information. On the other hand, obtaining information from internet news sites and search engines, governments, or friends or family members was associated with lower risk perception of COVID-19 infection. It was also lower among those who had higher trust in television, radio, newspapers and governments as sources of information. The variability in results across information sources supports the scientific finding that risk communication, which is the basis for accurate and scientific risk perception, should be implemented carefully (Aakko, 2004; Keller & Siegrist, 2009), and that ineffective risk communication can lead to misperceptions and health behaviors that further hinder the effectiveness of risk management (Zhang et al., 2020).

### 4.2 Factors associated with risk perception of severe illness within the next 6 months

Contrary to the risk perception of infection, age was statistically associated with higher risk perception of severe illness. The fact that the relationship between certain risk factors and COVID-19 risk perception differed between becoming infected and becoming severely ill suggests that individuals have different reasons for coming to their epistemological positions (Hornsey et al., 2018). This finding of a relationship between age and perceived risk of COVID-19 severity is also consistent with previous studies (Bruine de Bruin, 2021; Laires et al., 2021). As mentioned above, in a study conducted in 2020 in the United States, older people had a higher perceived risk of dying from infection and a lower perceived risk of infection (Bruine de Bruin, 2021). This may be related to the fact that the population is aware of the evidence that older age is a risk factor for severe COVID-19 infection (Fang et al., 2020, Gallo Marin et al., 2021). The same reason may be at play in the present study, in which the presence of underlying diseases was statistically significantly associated with higher risk perception of severe illness. Many studies have reported that those with comorbidities are a high-risk group for COVID-19 severe illness (Cook, 2020; Hussain et al., 2020; Kalligeros et al., 2020; Laires et al., 2021; Shahid et al., 2020; Wang et al., 2020).

Similar to the results of risk perception of infection, those who perceive less risk from the COVID-19 vaccine and those who perceive more benefit from the vaccine were less likely to perceive the risk of severe illness. Many studies reported that COVID-19 vaccines were effective not only in preventing infection but also in lowering the risk of hospitalization and severe illness from COVID-19 (Britton et al., 2021, Public Health; Public Health England, 2021; Rinott et al., 2021). The participants’ awareness of those findings through certain information sources may have affected their risk perception of infection and severe illness.

Similar to the results for infection risk perception, those who used internet news sites and search engines as information sources about COVID-19 were more likely to have a lower risk perception of severe illness. In addition, the higher the trust in the government as a source of information, the lower the perceived risk of serious illness. On the other hand, television, radio and newspapers had the opposite result: the higher the trust, the higher the risk of serious illness. This may be due to the high intensity of information dissemination about the risk of serious illness on television and radio, but the reasons for this need to be further evaluated in detail.

A higher trust level in SNS as a COVD-19 information source was also associated with higher risk perception of severe illness. It is known that risk perception may be driven by emotion and feeling (Dillard et al., 2012; Sheeran et al., 2014; Slovic et al., 2004). It has been also pointed out that excessive use of SNS can influence people's emotions and fuel fear and anxiety, and these are likely to make people's risk perception more serious (Ali et al., 2019; Paek et al., 2016, Zeballos Rivas, Lopez Jaldin, Nina Canaviri, Portugal Escalante, Alanes Fernandez, et al., 2021). For example, a study that examined the relationship between exposure to COVID-19 information and psychological status among medical students in China in April 2020 showed that the higher the use of SNS, the higher the perceived risk of COVID-19 severe illness (Lin, Tu, & Beitsch, 2020). In addition, it was possible that participants obtained fake news about COVID-19 or the vaccine from SNS, and further research is needed to evaluate the association between risk perception and using SNS (Naeem et al., 2021; Orso et al., 2020).

### 4.3 Implications

Under emergency situations such as the COVID-19 pandemic, public authorities and scientists have a responsibility to disseminate public health messages (Pan American Health Organization, 2020; Sauer et al., 2021). The intensity of the COVID-19 preventive measures and activity restrictions required and the risks to watch out for (infection itself or severe illness) vary depending on the pandemic situation, vaccination coverage and vaccine effectiveness, and the development and spread of therapeutic agents. Depending on these circumstances, public health messages about the effectiveness and importance of measures and restrictions (including why they are necessary) should be disseminated to populations with the relevant sociodemographic or psychological and vaccine-related characteristics identified in our and other studies.

Although the speed of communication with the population and trust levels vary depending on the information source and the characteristics of the information, in an emergency situation such as the COVID-19 pandemic, it is important that scientifically accurate information is communicated without delay (Kim et al., 2021). In this study, we found that risk perception was related to information sources and trust levels in them. Tagini et al. has pointed out that risk perception increases with the use of and trust in disease-related information sources (Tagini, Brugnera, Ferrucci, Mazzocco, Compare, et al., 2021). This indicates that there are differences in the way a message is delivered (content and intensity) depending on the medium people use to access it, and further research is needed to evaluate these differences. For example, it is known that information sources such as SNS and television have very different user characteristics (Chen et al., 2018; Miller & West, 2007), and further research investigation is necessary on how messages can be effectively delivered to different user groups depending on the information source in order to maintain and improve the practice of preventive measures and adherence to COVID-19-related lifestyle restrictions.

### 4.4 Limitations

First, self-selection bias may have affected this study: participants may have been biased toward those who were interested in this research topic. Next, sampling bias, which often occurs in online surveys, may have also played a role (Szolnoki & Hoffmann, 2013). In this study, the distribution of age, gender, and prefecture population ratios was similar to the distribution of the total population as a result of the quota sampling method, but we did not adjust our sample with respect to educational status, which may have affected the results. Another common bias of online surveys is that participants are limited to those with access to the Internet and digital devices. This could be a significant bias, especially with respect to the older population (Hargittai et al., 2019). As a methodological limitation, the COVID-19 infection status in each respondent's place of residence, which would uniquely correspond to the residence variable, could not be considered in the models as an adjustment variable. Thus, it should be noted that the possible regional differences in risk perception of COVID-19 indicated by the estimation results for the place of residence variable include not only region-specific differences but also differences in COVID-19 infection status. In addition, little information was available about the COVID-19 vaccine at the time of the survey, which may have influenced the participants' knowledge and attitudes about the COVID-19 vaccine. Because the survey included the item “already infected” as one of the options for the question measuring risk perception, those who had already been infected were excluded from this study, and we could not assess their risk perception. Finally, as this was a cross-sectional study, it is difficult to make conclusions regarding causation.

## 5 Conclusion

The results of this study, based on the largest survey regarding COVID-19 in Japan, provide important evidence of factors associated with the risk perception of COVID-19 infection and severe illness. Risk perceptions were associated with sociodemographic and vaccine-related factors, as well as with the source of COVID-19 information and corresponding trust levels in those sources. Our findings indicate the importance of identifying, understanding and addressing such factors to better communicate public health messages for maintaining and improving the practice of preventive measures and adherence to COVID-19-related lifestyle restrictions.

## Ethics approval

Ethical approval was granted by the Ethics Committee of Keio University School of Medicine under authorization number 20200340.

## Author statement

Megumi Adachi: Conceptualization; Methodology; Software; Validation; Formal analysis; Investigation; Writing - Original Draft; Writing - Review & Editing; Visualization. Michio Murakami: Conceptualization; Methodology; Validation; Investigation; Writing - Original Draft; Writing - Review & Editing; Visualization. Daisuke Yoneoka: Conceptualization; Methodology; Software; Validation; Formal analysis; Investigation; Writing - Original Draft; Writing - Review & Editing. Takayuki Kawashima: Conceptualization; Methodology; Software; Validation; Formal analysis; Investigation; Writing - Original Draft; Writing - Review & Editing. Masahiro Hashizume: Methodology; Validation; Writing - Original Draft; Writing - Review & Editing; Supervision. Haruka Sakamoto: Methodology; Investigation; Writing - Original Draft; Writing - Review & Editing. Akifumi Eguchi: Conceptualization; Methodology; Software; Validation; Formal analysis; Investigation; Writing - Original Draft; Writing - Review & Editing. Cyrus Ghaznavi: Investigation; Writing - Original Draft; Writing - Review & Editing. Stuart Gilmour: Methodology; Validation; Writing - Original Draft; Writing - Review & Editing. Satoshi Kaneko: Conceptualization; Validation; Investigation; Resources; Data Curation; Writing - Original Draft; Writing - Review & Editing; Visualization; Supervision. Hiroyuki Kunishima: Conceptualization; Validation; Investigation; Resources; Data Curation; Writing - Original Draft; Writing - Review & Editing; Visualization; Supervision. Keiko Maruyama-Sakurai: Investigation; Resources; Data Curation; Writing - Original Draft; Writing - Review & Editing; Visualization; Supervision. Yuta Tanoue: Conceptualization; Methodology; Software; Validation; Formal analysis; Investigation; Writing - Original Draft; Writing - Review & Editing. Yoshiko Yamamoto: Methodology; Validation; Writing - Original Draft; Writing - Review & Editing. Hiroaki Miyata: Conceptualization; Validation; Investigation; Resources; Data Curation; Writing - Original Draft; Writing - Review & Editing; Visualization; Supervision; Project administration; Funding acquisition. Shuhei Nomura: Conceptualization; Methodology; Software; Validation; Formal analysis; Investigation; Resources; Data Curation; Writing - Original Draft; Writing - Review & Editing; Visualization; Supervision; Project administration; Funding acquisition.

## Funding

This work was partly funded by research grants from the Ministry of Health, Labour and Welfare of Japan (H29-Gantaisaku-ippan-009), the 10.13039/100009619Japan Agency for Medical Research and Development (AMED) (JP20fk0108535), and the 10.13039/501100001700Ministry of Education, Culture, Sports, Science and Technology of Japan (21H03203).

## Consent to participate

Respondents had to provide their consent before they proceeded to the questionnaire response page.

## Consent for publication

Not applicable.

## Availability of data and material

The datasets generated during and/or analyzed during the current study are not publicly available due to ethical considerations but are available from the corresponding author on reasonable request.

## Code availability

Not applicable.

## Declaration of competing interest

The authors declare that they have no competing interests.

## References

[bib1] Aakko E. (2004). Risk communication, risk perception, and public health. Wmj.

[bib2] Af WÅhlberg A.E. (2001). The theoretical features of some current approaches to risk perception. Journal of Risk Research.

[bib3] Ali K., Zain-ul-abdin K., Li C., Johns L., Ali A.A., Carcioppolo N. (2019). Viruses going viral: Impact of fear-arousing sensationalist social media messages on user engagement. Science Communication.

[bib4] Bar-On Y.M., Goldberg Y., Mandel M., Bodenheimer O., Amir O., Freedman L. (2022). Protection by a fourth dose of BNT162b2 against omicron in Israel. New England Journal of Medicine.

[bib5] Barr M., Raphael B., Taylor M., Stevens G., Jorm L., Giffin M. (2008). Pandemic influenza in Australia: Using telephone surveys to measure perceptions of threat and willingness to comply. BMC Infectious Diseases.

[bib6] Bowling A. (2005). Just one question: If one question works, why ask several?. Journal of Epidemiology & Community Health.

[bib7] Brewer N.T., Chapman G.B., Gibbons F.X., Gerrard M., McCaul K.D., Weinstein N.D. (2007). Meta-analysis of the relationship between risk perception and health behavior: The example of vaccination. Health Psychology.

[bib8] Britton A., Jacobs Slifka K.M., Edens C., Nanduri S.A., Bart S.M., Shang N. (2021). Effectiveness of the Pfizer-BioNTech COVID-19 vaccine among residents of two skilled nursing facilities experiencing COVID-19 outbreaks - Connecticut, December 2020-February 2021. MMWR Morb Mortal Wkly Rep.

[bib9] Bruine de Bruin W. (2021). Age differences in COVID-19 risk perceptions and mental health: Evidence from a national U.S. survey conducted in March 2020. Journals of Gerontology Series B: Psychological Sciences and Social Sciences.

[bib10] Carstensen L.L., Pasupathi M., Mayr U., Nesselroade J.R. (2000). Emotional experience in everyday life across the adult life span. Journal of Personality and Social Psychology.

[bib11] Centers for Disease Control and Prevention (CDC) COVID data tracker;. https://covid.cdc.gov/covid-data-tracker/#trends_dailycases.

[bib12] Centers for Disease Control and Prevention (CDC) (2021). COVID-19 vaccine booster shots. https://www.cdc.gov/coronavirus/2019-ncov/vaccines/booster-shot.html.

[bib13] Chan E.Y.Y., Huang Z., Lo E.S.K., Hung K.K.C., Wong E.L.Y., Wong S.Y.S. (2020). Sociodemographic predictors of health risk perception, attitude and behavior practices associated with health-emergency disaster risk management for biological hazards: The case of COVID-19 pandemic in Hong Kong, SAR China. International Journal of Environmental Research and Public Health.

[bib14] Chen X., Hay J.L., Waters E.A., Kiviniemi M.T., Biddle C., Schofield E. (2018). Health literacy and use and trust in health information. Journal of Health Communication.

[bib15] Chisty M.A., Islam M.A., Munia A.T., Rahman M.M., Rahman N.N., Mohima M. (2021). Risk perception and information-seeking behavior during emergency: An exploratory study on COVID-19 pandemic in Bangladesh. International Journal of Disaster Risk Reduction.

[bib16] Cook T.M. (2020). The importance of hypertension as a risk factor for severe illness and mortality in COVID-19. Anaesthesia.

[bib17] Cross Marketing Inc Cross marketing. https://www.cross-m.co.jp.

[bib18] Dillard A.J., Ferrer R.A., Ubel P.A., Fagerlin A. (2012). Risk perception measures' associations with behavior intentions, affect, and cognition following colon cancer screening messages. Health Psychology.

[bib19] Ding Y., Du X., Li Q., Zhang M., Zhang Q., Tan X. (2020). Risk perception of coronavirus disease 2019 (COVID-19) and its related factors among college students in China during quarantine. PLoS One.

[bib20] Douglas M.M., Wildavsky A. (1983).

[bib21] Dryhurst S., Schneider C.R., Kerr J., Freeman A.L.J., Recchia G., van der Bles A.M. (2020). Risk perceptions of COVID-19 around the world. Journal of Risk Research.

[bib22] Eyeberu A., Mengistu D.A., Negash B., Alemu A., Abate D., Raru T.B. (2021). Community risk perception and health-seeking behavior in the era of COVID-19 among adult residents of Harari regional state, eastern Ethiopia. SAGE Open Medicine.

[bib23] Fang X., Li S., Yu H., Wang P., Zhang Y., Chen Z. (2020). Epidemiological, comorbidity factors with severity and prognosis of COVID-19: A systematic review and meta-analysis. Aging (Albany NY).

[bib24] Gallo Marin B., Aghagoli G., Lavine K., Yang L., Siff E.J., Chiang S.S. (2021). Predictors of COVID-19 severity: A literature review. Reviews in Medical Virology.

[bib25] Hargittai E., Piper M.A., Morris R.M. (2019). Fom internet access to internet skills: Digital inequality among older adults. Universal Access in the Information Society.

[bib26] He S., Chen S., Kong L., Liu W. (2021). Analysis of risk perceptions and related factors concerning COVID-19 epidemic in Chongqing, China. Journal of Community Health.

[bib27] Hornsey M.J., Harris E.A., Fielding K.S. (2018). The psychological roots of anti-vaccination attitudes: A 24-nation investigation. Health Psychology.

[bib28] Hussain A., Bhowmik B., do Vale Moreira N.C. (2020). COVID-19 and diabetes: Knowledge in progress. Diabetes Research and Clinical Practice.

[bib29] Institute of Medicine (2012).

[bib30] Joffe H. (2003). Risk: From perception to social representation. British Journal of Social Psychology.

[bib31] Kalam M.A., Davis T.P., Shano S., Uddin M.N., Islam M.A., Kanwagi R. (2021). Exploring the behavioral determinants of COVID-19 vaccine acceptance among an urban population in Bangladesh: Implications for behavior change interventions. PLoS One.

[bib32] Kalligeros M., Shehadeh F., Mylona E.K., Benitez G., Beckwith C.G., Chan P.A. (2020). Association of obesity with disease severity among patients with coronavirus disease 2019. Obesity.

[bib33] Kasperson R.E., Renn O., Slovic P., Brown H.S., Emel J., Goble R. (1988). The social amplification of risk: A conceptual framework. Risk Analysis.

[bib34] Keller C., Siegrist M. (2009). Effect of risk communication formats on risk perception depending on numeracy. Medical Decision Making.

[bib35] Kim S., Capasso A., Cook S.H., Ali S.H., Jones A.M., Foreman J. (2021). Impact of COVID-19-related knowledge on protective behaviors: The moderating role of primary sources of information. PLoS One.

[bib36] Laires P.A., Dias S., Gama A., Moniz M., Pedro A.R., Soares P. (2021). The association between chronic disease and serious COVID-19 outcomes and its influence on risk perception: Survey study and database analysis. JMIR Public Health Surveill.

[bib37] Leiserowitz A. (2006). Climate change risk perception and policy preferences: The role of affect, imagery, and values. Climatic Change.

[bib38] Lim V.W., Lim R.L., Tan Y.R., Soh A.S., Tan M.X., Othman N.B. (2021). Government trust, perceptions of COVID-19 and behaviour change: Cohort surveys, Singapore. Bulletin of the World Health Organization.

[bib39] Lin Y., Hu Z., Alias H., Wong L.P. (2020). Influence of mass and social media on psychobehavioral responses among medical students during the downward trend of COVID-19 in Fujian, China: Cross-sectional study. Journal of Medical Internet Research.

[bib40] Lin C., Tu P., Beitsch L.M. (2020). Confidence and receptivity for COVID-19 vaccines: A rapid systematic review. Vaccines.

[bib41] Loewenstein G.F., Weber E.U., Hsee C.K., Welch N. (2001). Risk as feelings. Psychological Bulletin.

[bib42] Mansilla Domínguez J.M., Font Jiménez I., Belzunegui Eraso A., Peña Otero D., Díaz Pérez D., Recio Vivas A.M. (2020). Risk perception of COVID-19 community transmission among the Spanish population. International Journal of Environmental Research and Public Health.

[bib43] Miller E.A., West D.M. (2007). Characteristics associated with use of public and private web sites as sources of health care information: Results from a national survey. Medical Care.

[bib44] Ministry of Health Back to life vaccines for coronavirus. https://govextra.gov.il/ministry-of-health/covid19-vaccine/en-covid-19-vaccine-3rd-dose/.

[bib45] Monge-Rodríguez F.S., Jiang H., Zhang L., Alvarado-Yepez A., Cardona-Rivero A., Huaman-Chulluncuy E. (2021). Psychological factors affecting risk perception of COVID-19: Evidence from Peru and China. International Journal of Environmental Research and Public Health.

[bib46] Myhre A., Xiong T., Vogel R.I., Teoh D. (2020). Associations between risk-perception, self-efficacy and vaccine response-efficacy and parent/guardian decision-making regarding adolescent HPV vaccination. Papillomavirus Research.

[bib47] Naeem S.B., Bhatti R., Khan A. (2021). An exploration of how fake news is taking over social media and putting public health at risk. Health Information and Libraries Journal.

[bib48] National Health Service (NHS) Coronavirus (COVID-19) vaccine 3rd dose. https://www.nhs.uk/conditions/coronavirus-covid-19/coronavirus-vaccination/coronavirus-vaccine-3rd-dose/.

[bib49] Neubauer A.B., Smyth J.M., Sliwinski M.J. (2019). Age differences in proactive coping with minor hassles in daily life. Journals of Gerontology Series B: Psychological Sciences and Social Sciences.

[bib50] Nomura S., Eguchi A., Yoneoka D., Kawashima T., Tanoue Y., Murakami M. (2021). Reasons for being unsure or unwilling regarding intention to take COVID-19 vaccine among Japanese people: A large cross-sectional national survey. Lancet Reg Health West Pacific.

[bib51] Omer S.B., Benjamin R.M., Brewer N.T., Buttenheim A.M., Callaghan T., Caplan A. (2021). Promoting COVID-19 vaccine acceptance: Recommendations from the lancet commission on vaccine refusal, acceptance, and demand in the USA. Lancet.

[bib52] Orso D., Federici N., Copetti R., Vetrugno L., Bove T. (2020). Infodemic and the spread of fake news in the COVID-19-era. European Journal of Emergency Medicine.

[bib53] (2020). Our world in data. COVID-19 data exploer. https://ourworldindata.org/explorers/coronavirus-data-explorer?zoomToSelection=true&facet=none&pickerSort=desc&pickerMetric=total_vaccinations_per_hundred&Metric=People+fully+vaccinated&Interval=Cumulative&Relative+to+P.

[bib54] Paek H.J., Oh S.H., Hove T. (2016). How fear-arousing news messages affect risk perceptions and intention to talk about risk. Health Communication.

[bib55] Pan American Health Organization (2020).

[bib56] Paulik L.B., Keenan R.E., Durda J.L. (2020). The case for effective risk communication: Lessons from a global pandemic. Integrated Environmental Assessment and Management.

[bib57] Public Health England (2021).

[bib58] Public Health England. GOV UK coronavirus (COVID-19) in the UK. https://coronavirus.data.gov.uk.

[bib59] Rinott E., Youngster I., Lewis Y.E. (2021). Reduction in COVID-19 patients requiring mechanical ventilation following implementation of a national COVID-19 vaccination program - Israel, December 2020-February 2021. MMWR Morb Mortal Wkly Rep.

[bib60] Robinson E., Jones A., Lesser I., Daly M. (2021). International estimates of intended uptake and refusal of COVID-19 vaccines: A rapid systematic review and meta-analysis of large nationally representative samples. Vaccine.

[bib61] Sauer M.A., Truelove S., Gerste A.K., Limaye R.J. (2021). A failure to communicate? How public messaging has strained the COVID-19 response in the United States. Health Security.

[bib62] Setbon M., Raude J. (2010). Factors in vaccination intention against the pandemic influenza A/H1N1. The European Journal of Public Health.

[bib63] Shahid Z., Kalayanamitra R., McClafferty B., Kepko D., Ramgobin D., Patel R. (2020). COVID-19 and older adults: What we know. Journal of the American Geriatrics Society.

[bib64] Sheeran P., Harris P.R., Epton T. (2014). Does heightening risk appraisals change people's intentions and behavior? A meta-analysis of experimental studies. Psychological Bulletin.

[bib65] Shiina A., Niitsu T., Kobori O., Idemoto K., Hashimoto T., Sasaki T. (2020). Relationship between perception and anxiety about COVID-19 infection and risk behaviors for spreading infection: A national survey in Japan. Brain Behaviour Immunology Health.

[bib66] Sjöberg L. (2002). Are received risk perception models alive and well?. Risk Analysis.

[bib67] Slovic P. (1987). Perception of risk. Science.

[bib68] Slovic P. (2010).

[bib69] Slovic P., Finucane M.L., Peters E., MacGregor D.G. (2004). Risk as analysis and risk as feelings: Some thoughts about affect, reason, risk, and rationality. Risk Analysis.

[bib70] Slovic P., Fischhoff B., Lichtenstein S. (1982). Why study risk perception?. Risk Analysis.

[bib71] Szolnoki G., Hoffmann D. (2013). Online, face-to-face and telephone surveys–comparing different sampling methods in wine consumer research. Wine Economics and Policy.

[bib72] Tagini S., Brugnera A., Ferrucci R., Mazzocco K., Compare A., Silani V. (2021). It won't happen to me! Psychosocial factors influencing risk perception for respiratory infectious diseases: A scoping review. Application Psychology Health Well Being.

[bib73] Tagini S., Brugnera A., Ferrucci R., Mazzocco K., Pievani L., Priori A. (2021). Attachment, personality and locus of Control: Psychological determinants of risk perception and preventive behaviors for COVID-19. Frontiers in Psychology.

[bib74] Tartof S.Y., Slezak J.M., Fischer H., Hong V., Ackerson B.K., Ranasinghe O.N. (2021). Effectiveness of mRNA BNT162b2 COVID-19 vaccine up to 6 months in a large integrated health system in the USA: A retrospective cohort study. Lancet.

[bib75] Van der Linden S. (2015). The social-psychological determinants of climate change risk perceptions: Towards a comprehensive model. Journal of Environmental Psychology.

[bib76] Van der Linden S. (2017).

[bib77] Wang B., Li R., Lu Z., Huang Y. (2020). Does comorbidity increase the risk of patients with COVID-19: Evidence from meta-analysis. Aging (Albany NY).

[bib78] Weinstein N.D., Kwitel A., McCaul K.D., Magnan R.E., Gerrard M., Gibbons F.X. (2007). Risk perceptions: Assessment and relationship to influenza vaccination. Health Psychology.

[bib79] Wildavsky A., Dake K. (1990). Theories of risk perception: Who fears what and why?. Daedalus [Internet].

[bib80] World Health Organization (2018).

[bib81] World Health Organization (2020). COVID-19 dashbord (Israel). https://covid19.who.int/region/euro/country/il.

[bib82] World Health Organization (2020). COVID-19 vaccines. https://www.who.int/emergencies/diseases/novel-coronavirus-2019/covid-19-vaccines.

[bib83] World Health Organization (2020). WHO coronavirus (COVID-19) dashboard. https://covid19.who.int.

[bib84] Zeballos Rivas D.R., Lopez Jaldin M.L., Nina Canaviri B., Portugal Escalante L.F., Alanes Fernandez A.M.C., Aguilar Ticona J.P. (2021). Social media exposure, risk perception, preventive behaviors and attitudes during the COVID-19 epidemic in La paz, Bolivia: A cross sectional study. PLoS One.

[bib85] Zeballos Rivas D.R., Lopez Jaldin M.L., Nina Canaviri B., Portugal Escalante L.F., Alanes Fernández A.M.C., Aguilar Ticona J.P. (2021). Social media exposure, risk perception, preventive behaviors and attitudes during the COVID-19 epidemic in La paz, Bolivia: A cross sectional study. PLoS One.

[bib86] Zhang L., Li H., Chen K. (2020). Effective risk communication for public health emergency: Reflection on the COVID-19 (2019-nCoV) outbreak in Wuhan, China. Healthcare (Basel).

[bib87] de Zwart O., Veldhuijzen I.K., Elam G., Aro A.R., Abraham T., Bishop G.D. (2007). Avian influenza risk perception, Europe and Asia. Emerging Infectious Diseases.

